# Systemic CD4 immunity: A powerful clinical biomarker for PD‐L1/PD‐1 immunotherapy

**DOI:** 10.15252/emmm.202012706

**Published:** 2020-07-10

**Authors:** Miren Zuazo, Hugo Arasanz, Ana Bocanegra, Luisa Chocarro, Ruth Vera, David Escors, Hiroshi Kagamu, Grazyna Kochan

**Affiliations:** ^1^ Navarrabiomed‐UPNA IdISNA Pamplona Spain; ^2^ Complejo Hospitalario de Navarra‐IdISNA Pamplona Spain; ^3^ Saitama Medical University International Medical Center Hidaka Japan

**Keywords:** Cancer, Immunology

## Abstract

The search for non‐invasive systemic biomarkers of response to PD‐L1/PD‐1 blockade immunotherapy is currently a priority in oncoimmunology. In contrast to classical tumor biomarkers, the identification of clinically useful immunological biomarkers is certainly a challenge, as anti‐cancer immune responses depend on the coordinated action of many cell types. Studies on the dynamics of systemic CD8 T‐cell populations have provided indications that such biomarkers may have a place in clinical practice. However, the power of CD8 T‐cell subsets to discriminate clinical responses in immunotherapy has so far proven to be limited. The systemic evaluation of CD8 T‐cell regulators such as myeloid cells and CD4 T cells may provide the solution. Here we discuss the value of systemic quantification of CD4 T‐cell subsets for patient selection in light of the results obtained by Prof. Kagamu′s and our team. Our studies have independently demonstrated that the evaluation of the pre‐treatment status of systemic CD4 immunity is a critical factor for the clinical outcome of PD‐L1/PD‐1 blockade therapy with robust predictive capacities.

## Graphical abstract

In this Commentary, D. Escors *et al* discuss the exciting possibility of using systemic CD4 immunity pre‐treatment status as a predictive tool of clinical outcome following PD‐L1/PD‐1 blockade cancer immunotherapy.

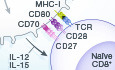

## The revolution of PD‐L1/PD‐1 blockade immunotherapy, but not for all

It comes as no surprise nowadays that PD‐L1/PD‐1 blockade immunotherapy has changed the treatment paradigm for several cancer types. Yet, a significant number of patients are refractory and the reasons behind this fact seem to be several. Nevertheless, it highlights the urgent necessity for accurate biomarkers of response for the identification of patients that will benefit from PD‐L1/PD‐1 blockade immunotherapies, and those that will progress or develop hyperprogressive disease. Moreover, the precise molecular mechanisms by which T‐cell functions are stimulated by PD‐L1/PD‐1 inhibitors remain to be fully understood. Such understanding is also relevant for designing approaches that increase clinical efficacy and overcome resistance to treatment.

## Effector CD8 T cells and their value as biomarkers of response

Tumor‐infiltrating CD8 T cells are the obvious initial “suspect” for a cellular “immunobiomarker” in immunotherapy. At the end of the day, these cells are the effector cytotoxic agents over cancer cells. However, there is some existing controversy on whether PD‐L1/PD‐1 inhibitors reinvigorate pre‐existing TIL immunity, or act over systemic T‐cell immunity by enhancing the expansion of novel T‐cell clonotypes that will subsequently infiltrate tumors (Scott *et al*, 2019; Yost *et al*, 2019). This may represent a significant difference regarding biomarkers, because the study of systemic immune cell populations from blood samples is marginally invasive, homogeneous and less costly than tumor sampling. Various groups have demonstrated a “proliferative burst” of systemic CD8 PD‐1^+^ T‐cell subpopulations after PD‐L1/PD‐1 blockade in several cancer types which correlate with clinical responses (Huang *et al*, 201v7; Kamphorst *et al*, 2017; Kim *et al*, 2019). For example, Kim *et al* (2019) quantified PD‐1^+^ CD8^+^ T‐cell expansion as a fold change in the percentage of Ki67^+^ cells (Ki‐67_D7/D0_ ≥ 2.8) after the first week of treatment in patients with thymic epithelial tumors treated with PD‐1 inhibitors. The predictive value of Ki‐67_D7/D0_ ≥ 2.8 was validated in two independent cohorts of non‐small cell lung cancer (NSCLC) patients. This is in agreement with a recent elegant study in melanoma patients showing rearrangements in the repertoire of peripheral memory cytotoxic CD8 T cells in responders to immune checkpoint blockade (Valpione *et al*, 2020). These studies support the quantification of proliferating peripheral PD‐1^+^ CD8 T‐cell subpopulations as a systemic biomarker of responses. CD8 T cells in this context can help clinicians on decision‐making in the early onset of the treatment, but do not identify deleterious adverse events such as hyperprogressive disease.

## Systemic CD4 T‐cell subsets as robust indicators of the efficacy of PD‐L1/PD‐1 blockade immunotherapies

**Figure 1:**
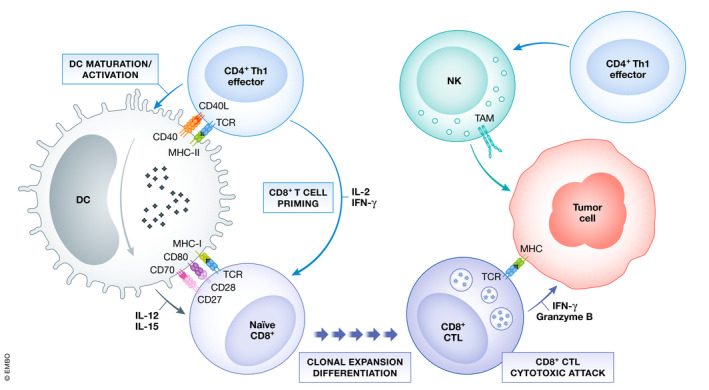
The contribution of CD4 Th1 subsets to anti‐tumor immunity The figure summarizes the well‐established roles of CD4 Th1 subsets in anti‐tumor responses. Right, CD4 Th1 cells allow the correct priming and differentiation of naive CD8 T into CTLs by secretion of cytokines and co‐stimulatory interactions with DCs within the secondary lymphoid organs. This process termed “DC licensing leads to DC maturation by CD40L‐CD40 binding. CD40‐CD40L signaling on DCs induces production of IL‐12 and IL‐15 and up‐regulates co‐stimulatory ligands CD80, CD86, and CD70, providing the required signals for CD8 CTL priming. CD80, CD86, and CD70 co‐stimulatory ligands on activated DC bind to their receptors CD28 and CD27 on naive CD8 T cells leading to CTL differentiation and survival. CD8 CTLs infiltrate tumors and exert cytotoxic responses against tumor cells after TAA recognition. Th1, T helper 1; CTL, cytotoxic T lymphocyte; DC, dendritic cell; TAA, tumor‐associated antigens.

It is a long‐standing fact that proficient CD8 anti‐tumor responses largely depend on CD4 help. However, this is often underestimated or at least overlooked by most studies on PD‐L1/PD‐1 blockade. CD4 T helper (Th) 1 cells promote the priming of effector and memory CD8 T cells by different mechanisms and interactions during antigen presentation/recognition (Borst *et al*, 2018) (Fig 1). Prof Kagamu′s and our team hypothesized that functional systemic CD4 immunity might be required to achieve efficacious CD8 responses in PD‐L1/PD‐1 blockade therapy.

Our studies have independently demonstrated that the pre‐treatment status of systemic CD4 immunity is a critical factor for clinical outcomes to PD‐L1/PD‐1 blockade therapy in NSCLC patients. Using different patient cohorts and analytical techniques, both studies found that equivalent baseline percentages of peripheral memory CD4 T cells strongly correlated with clinical benefit (Kagamu *et al*, 2019; Zuazo *et al*, 2019). In our study, we used conventional flow cytometry to quantify the relative percentages of systemic CD27^−^ CD28^low/−^ central and effector memory CD4 T cells as a potent discriminator of differential clinical outcomes (Zuazo *et al*, 2019). A cut‐off value of > 40% identified objective responders with an ORR 50%, while patients with < 40% showed an ORR of 0%, and were significantly associated with increased risk to hyperprogressive disease (Arasanz *et al*, 2020). Patients with low baseline numbers of memory CD4 T cells showed strong CD4 T‐cell dysfunctionality. Moreover, CD8 proliferative responses were only recovered in patients who had functional CD4 responses before treatment initiation. Kagamu *et al* simultaneously obtained similar conclusions through multiparametric flow cytometry identifying an equivalent CD62L^low^ effector memory CD4 T population which robustly discriminated patients with distinct clinical outcome, with similar threshold values found by us over systemic memory CD4 T cells. Hence, responders to PD‐1 blockade therapy had significantly higher baseline proportions of CD62L^low^ CD4 T cells, while non‐responders also had increased Tregs systemically. The study by Kagamu *et al* (2019) refined an algorithm that accounted for the ratio between memory CD4 and Treg cells to discriminate responders with a cut‐off value of ≥192 in two independent NSCLC cohorts. Gene expression analysis of CD62L^low^ CD4 T cells uncovered that these cells were TCR‐engaged proliferating Th1 cells, and up‐regulated pathways involved in the promotion of CD8 CTL responses.

## Conclusions and future directions

The future of CD4 T cells as systemic biomarkers of response to PD‐L1/PD‐1 blockade lies on more extensive validation cohorts for distinct cancer types, which is currently under way. The statistically powerful ROC analyses in our independent studies support its clinical application. In addition, each study used different phenotypic markers for the identification of CD4 memory populations. Therefore, unification of data provided by both studies will help us selecting the CD4 memory subpopulation with the strongest predictive value. Our working hypothesis is that CD4 memory help functions directly influence PD‐L1/PD‐1 efficacy over anti‐tumor CD8 responses. Nevertheless, the molecular mechanisms remain to be fully understood, but will very likely include antigen‐presenting cells such as dendritic cells. Finally, dynamic changes of CD4 T populations could help evaluate responses in “real‐time” from small blood samples during the duration of treatments. This will surely help identifying patients with a high risk of developing hyperprogression (Kagamu *et al*, 2019; Arasanz *et al*, 2020).

Concluding, strategies to restore CD4 immunity in patients before starting PD‐L1/PD‐1 blockade therapies could be part of the solution to minimize refractoriness.

## Conflict of interest

The authors declare that they have no conflict of interest.
